# Baseline T-lymphocyte and cytokine indices in sheep peripheral blood

**DOI:** 10.1186/s12917-022-03268-7

**Published:** 2022-05-05

**Authors:** Jihui Yang, Yongxue Lv, Yazhou Zhu, Shasha Li, Jia Tao, Liangliang Chang, Mingxing Zhu, Jiaqing Zhao, Yana Wang, Changyou Wu, Wei Zhao

**Affiliations:** 1 Center of Scientifc Technology of Ningxia Medical University, Yinchuan, China; 2 Ningxia Key Laboratory of Prevention and Treatment of Common Infectious Diseases, Yinchuan, China; 3 School of Basic Medical Science of Ningxia Medical University, Yinchuan, China; 4 Institute of Immunology, Zhongshan School of Medicine, Sun Yat-Sen University, Guangzhou, China

**Keywords:** Immunity, Peripheral blood, Sheep, T lymphocytes

## Abstract

**Background:**

Sheep are an important livestock species worldwide and an essential large-animal model for animal husbandry and veterinary research. Understanding fundamental immune indicators, especially T-lymphocyte parameters, is necessary for research on sheep diseases and vaccines, to better understand the immune response to bacteria and viruses for reducing the use of antibiotics and improving the welfare of sheep. We randomly selected 36 sheep of similar ages to analyze cell-related immune indicators in peripheral blood mononuclear cells (PBMCs). The proportions of CD4^+^ and CD8^+^ T cells in PBMCs were detected by flow cytometry. We used Concanavalin A (Con A) and Phorbol-12-myristate-13-acetate (PMA)/Ionomycin to stimulate PBMCs, and measured the expression of IFN-γ, IL-4, and IL-17A using enzyme-linked immunosorbent assay (ELISA) and enzyme-linked immunospot assay (ELISpot). Simultaneously, PMA/Ionomycin/brefeldin A (BFA) was added to PBMCs, then the expression of IFN-γ, IL-4, and IL-17A was detected by flow cytometry after 4 h of culturing. In addition, we observed the proliferation of PBMCs stimulated with Con A for 3, 4, and 5 days.

**Results:**

The proportions of CD4^+^ T lymphocytes (18.70 ± 4.21%) and CD8^+^ T lymphocytes (8.70 ± 3.65%) were generally consistent among individuals, with a CD4/CD8 ratio of 2.40 ± 0.79. PBMCs produced high levels of IFN-γ, IL-4, and IL-17A after stimulation with PMA/Ionomycin and Con A. Furthermore, PMA/Ionomycin stimulation of PBMC yielded significantly higher cytokine levels than Con A stimulation. Flow cytometry showed that the level of IFN-γ (51.49 ± 11.54%) in CD8^+^ T lymphocytes was significantly (*p* < 0.001) higher than that in CD4^+^ T lymphocytes (14.29 ± 3.26%); IL-4 (16.13 ± 6.81%) in CD4^+^ T lymphocytes was significantly (*p* < 0.001) higher than that in CD8^+^ T lymphocytes (1.84 ± 1.33%), There was no difference in IL-17A between CD4^+^ (2.83 ± 0.98%) and CD8^+^ T lymphocytes (1.34 ± 0.67%). The proliferation of total lymphocytes, CD4^+^ T lymphocytes, and CD8^+^ T lymphocytes continued to increase between days 3 and 5; however, there were no significant differences in proliferation between the cell types during the stimulation period.

**Conclusions:**

Evaluating primary sheep immune indicators, especially T lymphocytes, is significant for studying cellular immunity. This study provided valuable data and theoretical support for assessing the immune response of sheep to pathogens and improving sheep welfare.

## Background

Ruminants have been domesticated for over 10,000 years, providing meat, milk, and other auxiliary products indispensable for human survival in some areas [1, 2]. Zoonotic diseases like tuberculosis, listeriosis, tick-borne encephalitis, and transmissible spongiform encephalopathies pose significant threats to human health [3, 4]. Most of these diseases involve complex interactions between pathogens and host immune-system responses. These interactions and immune-related indicators need to be analyzed to develop safe and effective strategies for disease control.

Although small animals such as mice are standard biomedical models for studying many human and animal diseases, they cannot be used for studying ruminant-related diseases [1]. One of the most common ruminants, the sheep, is a suitable animal model owing to their size, availability, and ease of breeding. Sheep have been widely used as animal models to understand basic immune mechanisms, with satisfactory results [5–7]. It is necessary to measure the initial immune parameters when using sheep as a model in research on diseases and vaccines.

This study focused on detecting the ratio of CD4^+^ and CD8^+^ T cells in peripheral blood lymphocytes, the expression of several cytokines after stimulation with polyclonal stimulants, and the proliferation of stimulated lymphocytes in sheep. This information is essential for interpreting immunological mechanisms in sheep and improving sheep welfare.

## Results

### Proportions of CD4^+^ and CD8^+^ T lymphocytes in PBMCs

**Fig. 1:**
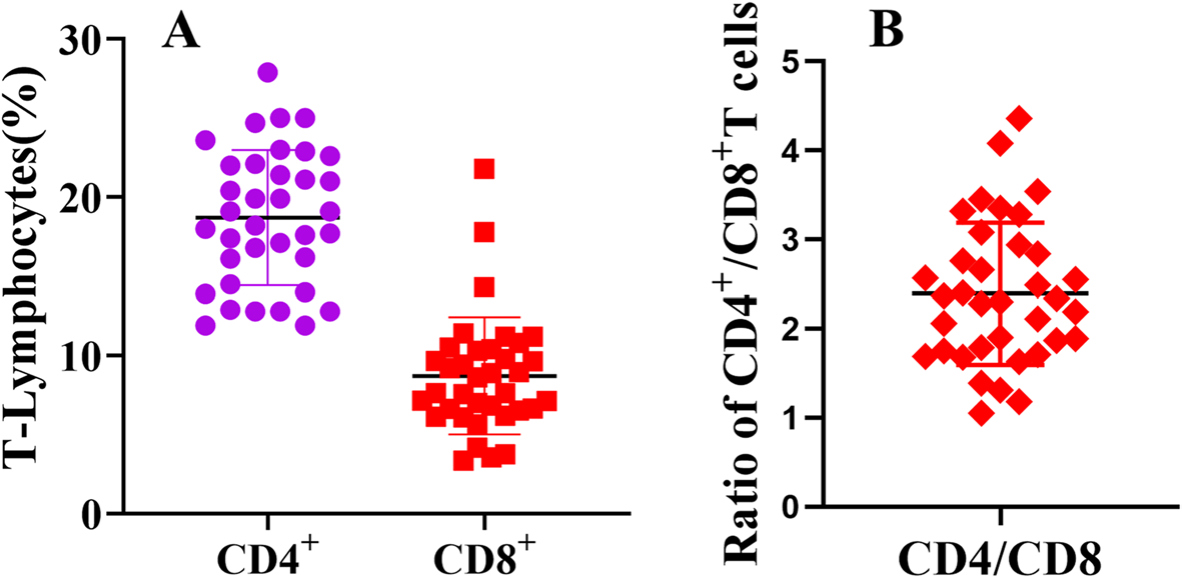
Populations of CD4^+^ T and CD8^+^ T lymphocytes in PBMCs. PBMC obtained from 36 sheep and stained with CD4 and CD8 antibodies were detected by flow cytometry. **A** Abundance of CD4^+^ and CD8^+^ T lymphocytes in PBMCs. **B** CD4/CD8 ratio. Levels and ratios are expressed as mean ± SD

PBMCs isolated from the peripheral blood were labeled with antibodies against CD4 and CD8 and analyzed by flow cytometry. After selecting the lymphocyte population and excluding adherent cells, we got the abundance of CD4^+^ T and CD8^+^ T lymphocytes. The mean CD4^+^ and CD8^+^ T lymphocyte proportions in the 36 samples were 18.70 ± 4.21% and 8.70 ± 3.65%, respectively (Fig. 1A); these data yielded a CD4/CD8 ratio of 2.40 ± 0.79 (Fig. 1B).

### Cytokine production by stimulated PBMCs in comparison with the medium control

**Fig. 2:**
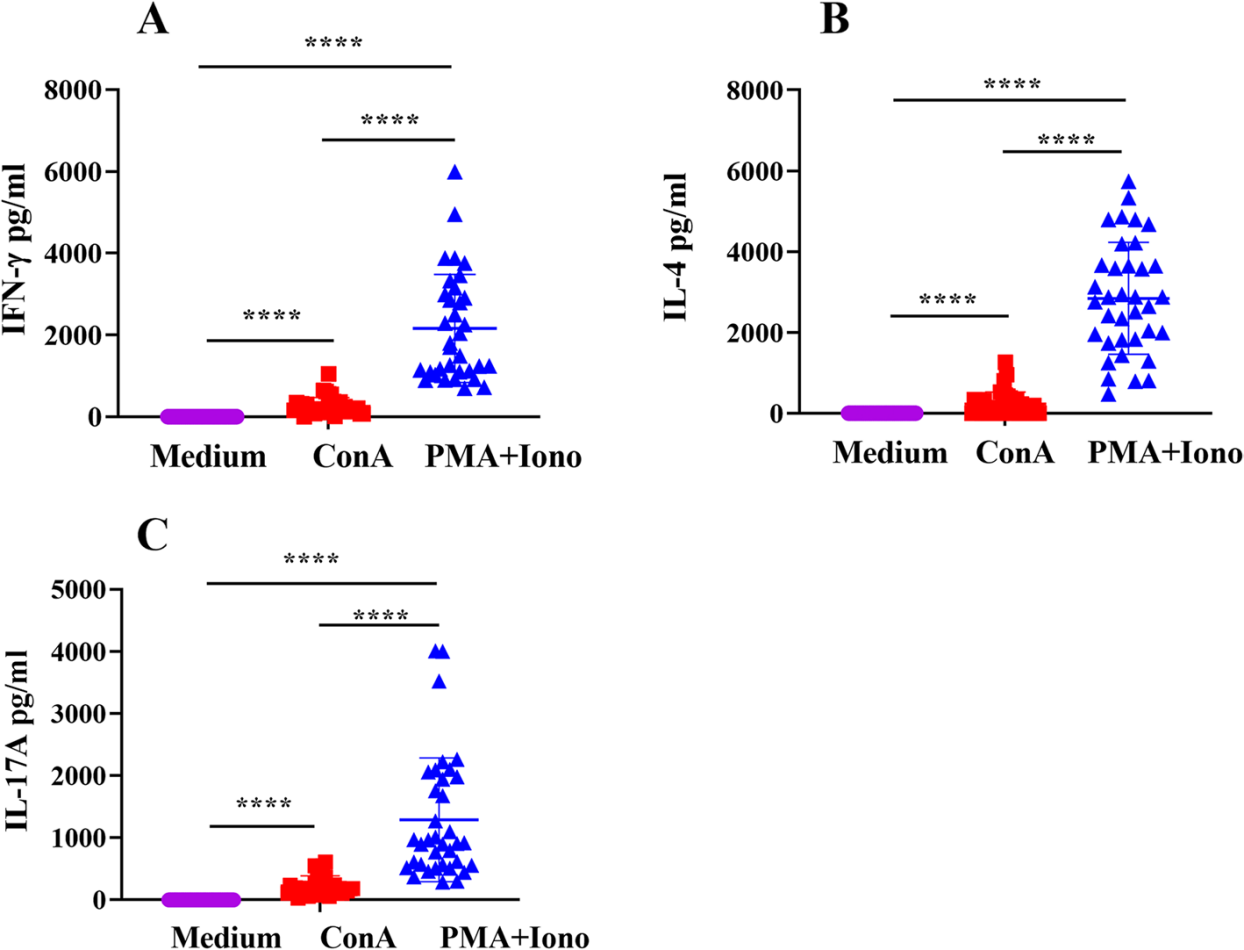
PBMC cytokine production in response to stimulation. PBMCs were added to U-bottom cell culture plates with Con A (5 μg/ml), PMA (20 ng/ml) + Ionomycin (1 μg/ml) or medium in triplicate for each sample and cultured at 37 °C and 5% CO_2_ for 24 h. After the incubation, the culture supernatant was collected, and cytokines were detected by ELISA. Optical density (OD) values from the spectrophotometer at 450 nm were used to construct a standard curve and quantify cytokine expression. **A** Expression of IFN-γ in PBMCs. **B** Expression of IL-4 in PBMCs. **C** Expression of IL-17A in PBMCs. Data are presented as means ± SD with statistically significant differences between stimulation groups (****p < 0.001)

High levels of cytokines were produced in the culture supernatant after stimulation with polyclonal stimulators PMA/Ionomycin and Con A, especially IFN-γ and IL-4 (Fig. 2A, B, C). After the stimulation with PMA/Ionomycin, the production of three cytokines IFN-γ, IL-4, and IL-17A, was significantly (*p* < 0.001) higher than that after the stimulation with Con A. The results indicated that PMA/Ionomycin stimulated the PBMCs to produce cytokines better than Con A.

### Enumeration of PBMCs expressing IFN-γ and IL-4 by ELISpot in comparison with the medium control

**Fig. 3:**
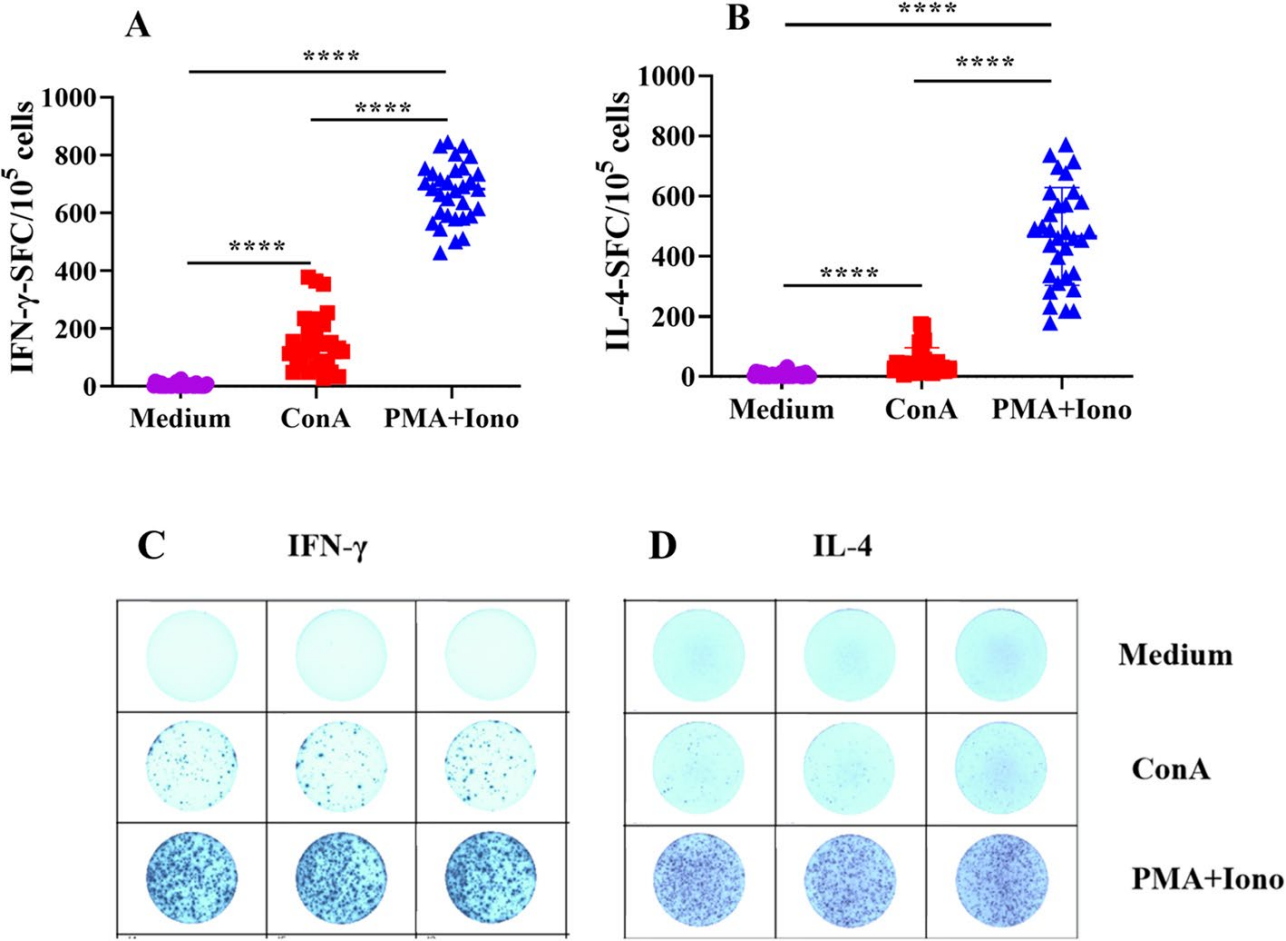
Detection of single-cell expression of IFN-γ and IL-4 by ELISpot. PBMCs were added to ELISpot plates in Con A (5 μg/ml), PMA (20 ng/ml) + Ionomycin (1 μg/ml), or medium in triplicate and cultured at 37 °C and 5% CO_2_ for 24 h. Spots were counted using an ELISpot reader system; mean numbers of spot-forming cells (SFCs) are shown per 10^5^ PBMCs. **A** SFC of IFN-γ stimulated under different conditions. **B** SFC of IL-4 stimulated under different conditions. **C** ELISpot representative images of IFN-γ activated with Con A and PMA + Ionomycin. **D** ELISpot representative images of IL-4 activated with Con A and PMA + Ionomycin. Data are presented as means ± SD with statistically significant differences between stimulation groups (****p < 0.001)

Consistent with the ELISA results, the two stimulants enhanced the production of a large number of IFN-γ and IL-4 -secreting cells by the PBMCs relative to the medium control (Fig. 3, *p* < 0.001). The average number of spots of IFN-γ and IL-4 in the PMA/Ionomycin activated PBMCs was significantly (*p* < 0.001) higher than that in Con A stimulated PBMCs (Fig. 3A, B). Figure 3C and D show technical replicates of PBMCs from the same sheep produced after stimulation by Con A or PMA/Ionomycin. The consistency of the technical repetition was visible, with almost no cells secreting IFN-γ or IL-4 in the medium control.

### Con A stimulation of PBMC proliferation over time in comparison with the medium control

**Fig. 4:**
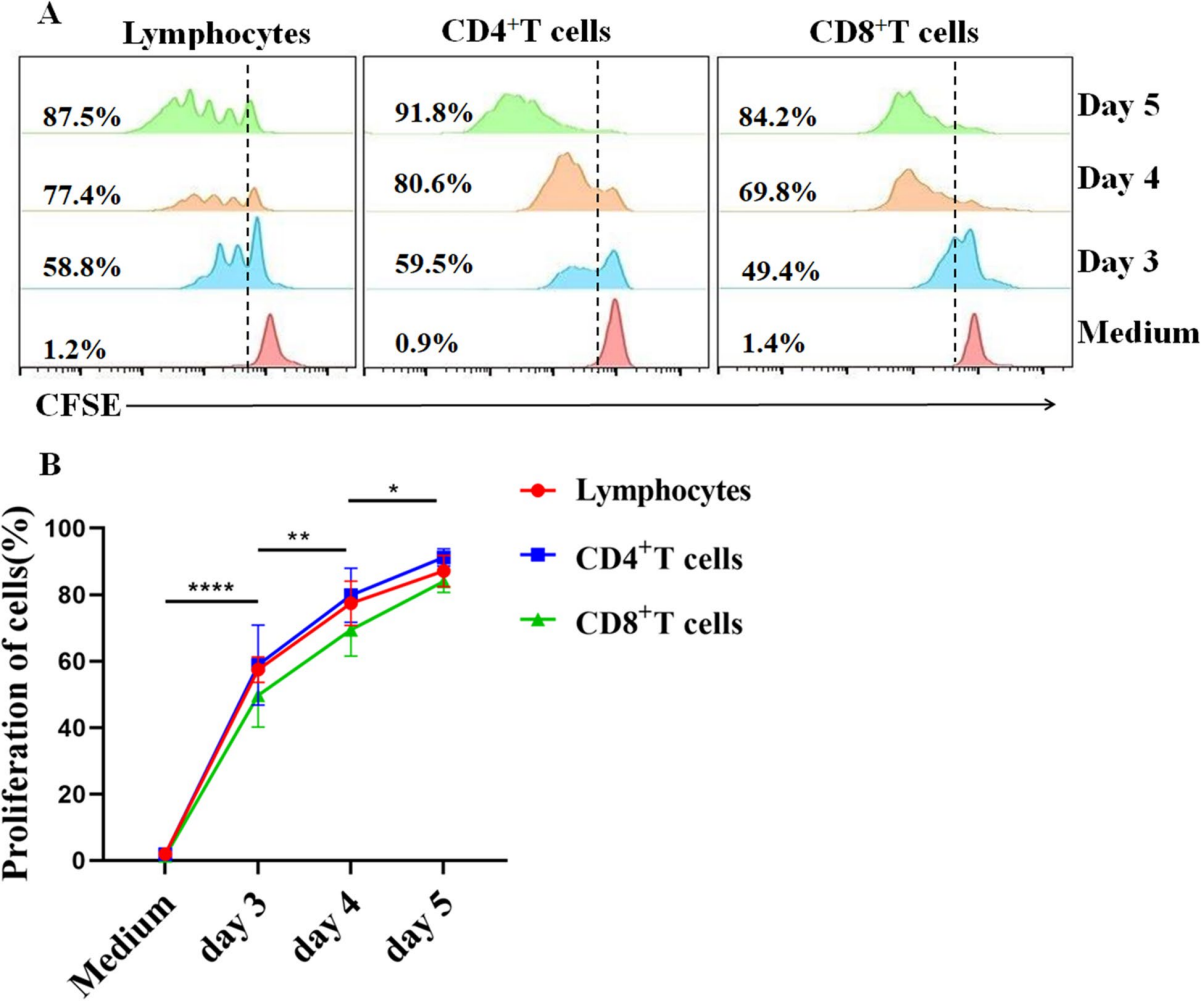
Con A-stimulated proliferation quantified by CFSE. PBMCs were stained with 5 μM CFSE at a concentration of 1 × 10^7^/ml and stimulated with Con A (5 μg/ml) or medium for 72, 96, and 120 h. Cells were collected and stained with mouse anti-sheep CD4 and CD8 antibodies. Samples were assayed by flow cytometry and data were analyzed using FlowJo software. **A** Representative graphs of the proliferation results of different cells at different times. **B** Proliferation statistics at different times. Statistically significant differences between stimulation times are indicated with asterisks (*p < 0.05, **p < 0.01, ****p < 0.001)

After 3 days of Con A stimulation of PBMCs, the number of lymphocytes, CD4^+^ T cells, and CD8^+^ T cells increased significantly compared with those in the medium control. The proportion of proliferating cells increased consistently from days 3 to 5 (Fig. 4A, B). In addition, there were no statistically significant differences in the proliferation of lymphocytes, CD4^+^ T cells, and CD8^+^ T cells.

### Expression of intracellular cytokines in PBMCs in comparison with the medium control

**Fig. 5:**
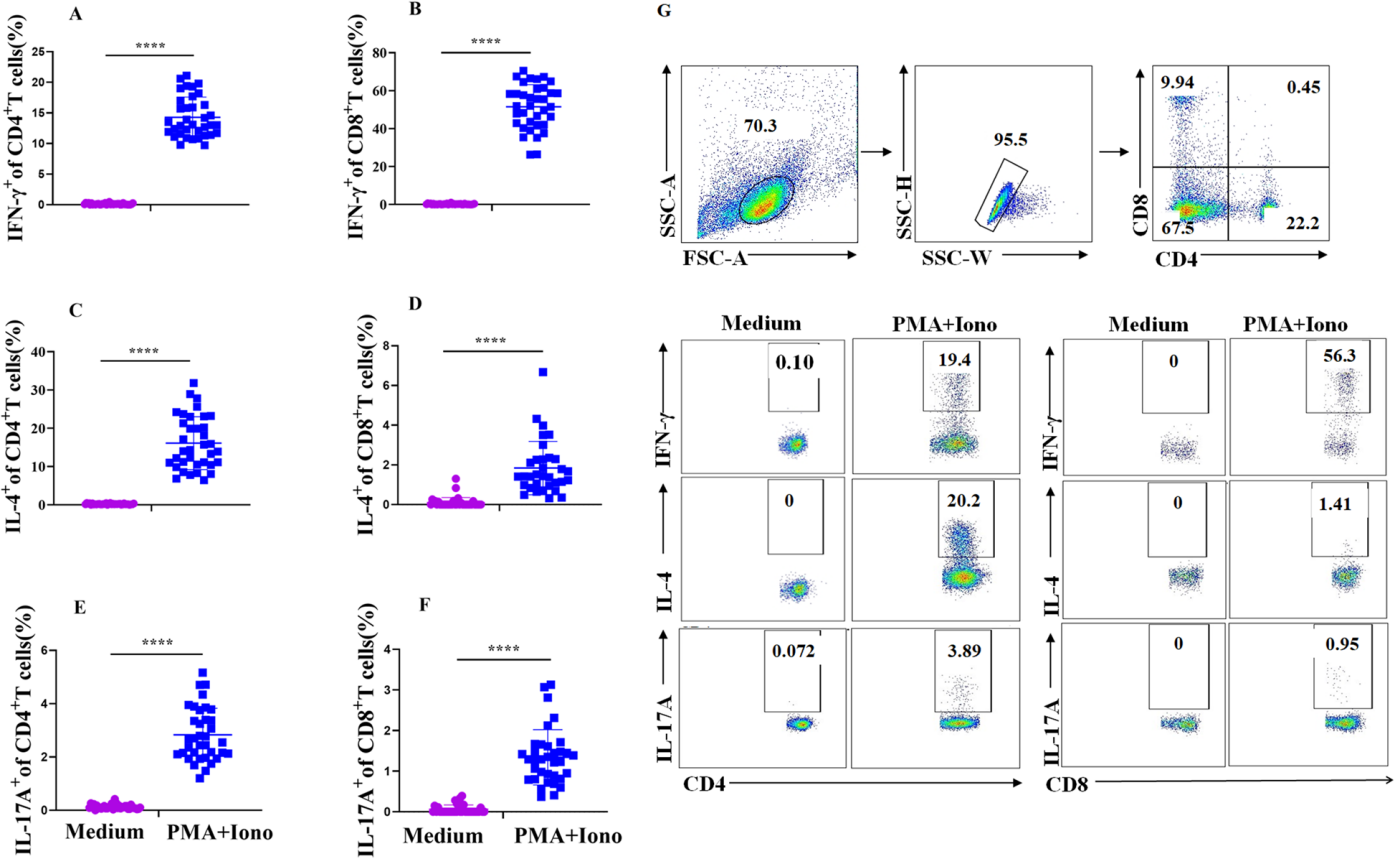
Intracellular cytokine expression in T cell subsets. PBMCs were stimulated with PMA (20 ng/mL)/Ionomycin (1 μg/mL)/BFA (1 μg/mL) or medium and cultured at 37 °C and 5% CO_2_ for 4 h. Cells were collected and stained with anti-CD4/CD8 antibodies and intracellular cytokine antibodies after being fixed and permeabilized. Data were acquired by flow cytometry and analyzed using FlowJo software. **A** Expression of IFN-γ in CD4^+^ T cells. **B** Expression of IFN-γ in CD8^+^ T cells. **C** Expression of IL-4 in CD4^+^ T cells. **D** Expression of IL-4 in CD8^+^ T cells. **E** Expression of IL-17A in CD4^+^ T cells. **F** Expression of IL-17A in CD8^+^ T cells. **G** Gating strategy for flow cytometry. Data are presented as means ± SD with statistically significant differences compared to the medium control (****p < 0.001)

PBMCs were collected, and flow cytometry detected intracellular cytokines after 4 h of stimulation with PMA/Ionomycin and BFA. Figure 5A and B show the expression of IFN-γ in CD4^+^ T (14.29 ± 3.26%) and CD8^+^ T (51.49% ± 11.54%) cells in activated PBMCs. The expression of IFN-γ in CD8^+^ T cells was significantly (*p* < 0.001) higher than that in CD4^+^ T cells. Similarly to IFN-γ, both CD4^+^ and CD8^+^ T cells produced high levels of IL-4 and IL-17A. However, the average level of IL-4 in CD4^+^ T cells (16.13 ± 6.81%) was higher than that in CD8^+^ T cells (1.84 ± 1.33%) (Fig. 5C, D). The expression of IL-17A in CD4^+^ T (2.83 ± 0.98%) and CD8^+^ T (1.34 ± 0.67%) cells increased significantly (*p* < 0.001) after PMA/Ionomycin stimulation compared with that in the medium control (Fig. 5E, F). The gating strategy used for flow cytometry is shown in Fig. 5G. We found no difference (*p* > 0.05) in the expression of IL-17A in CD4^+^ T and CD8^+^T cells.

## Discussion

Sheep are among the most important ruminants globally providing dairy and meat products necessary for human life [8–10]. Moreover, sheep are a widely used large-animal experimental model, which is significant for investigating multiple diseases and vaccines [7]. This model also provides insights into the immune response of sheep to bacteria and viruses, thereby reducing antibiotic use and improving sheep welfare [11, 12]. Our knowledge of a sheep’s immune function is not as extensive as that of other animals, such as mice. This requires us to conduct in-depth research on fundamental immune indicators, particularly those relevant to the functioning of T lymphocytes. However, no systematic reports have been published on the basic immune function indices of T lymphocytes in sheep. With the rapid development of immunological technology, the detection methods for immune-related indicators have become increasingly abundant and available. Immunoassay techniques such as ELISA, flow cytometry, and ELISpot experiments, each having unique detection advantages, have been used widely [13–16], individually or in combination to explain the immune response mechanism. This study assessed the abundance of CD4^+^ and CD8^+^ T lymphocytes in the peripheral blood of healthy sheep, together with cytokine production and the proliferation of PBMCs induced by multiple polyclonal stimulators PMA/Ionomycin and Con A.

T lymphocytes play an essential role in the immune response against infections, such as those caused by viruses and bacteria [17, 18]; both CD4^+^ and CD8^+^ T cells help in removing pathogens during infection [19, 20]. CD4^+^ T cells promote the production of antibodies by B cells, which are essential for producing memory CD8^+^ T cells and cytotoxicity. Bluetongue virus can elicit a T-cell response in sheep, mainly reflected as an increased abundance of both CD4^+^ and CD8^+^ T cells during the primary immune response [21–23]. The skin and local lymph nodes of sheep and goats show a strong immune and inflammatory response to pustule dermatitis virus infection, exhibiting cellular immune responses including CD4^+^ and CD8^+^ T cell changes in T cells [24]. Studies on virus infection in goats have shown that specific CD4^+^ T cells are the main mediators of protection against viral infection [25]. Immunization with *Echinococcus granulosus* myophilin in sheep can reduce the formation of cysts, partly because of the induction of cytokine production by T cells in the cellular immune response, suggesting that cytokines produced by T cells plays an important role in defense against parasitic infection [26]. We used flow cytometry to detect the proportions of CD4^+^ (Th) and CD8^+^ T (Tc) lymphocytes in PBMCs. Our research shows that the status of T lymphocytes in healthy sheep is similar, although there are some individual differences. The ratio of CD4/CD8^+^ T lymphocytes can reflect the level of immunity to a certain extent [27], indicating that the immune status of sheep is generally consistent. The existence of minor individual differences is understandable. This reiterates that when using sheep as experimental animals for related diseases or vaccine research, we should understand their immune status to evaluate the results more objectively. Gamma delta T cells are the main lymphocyte population in peripheral blood and inflammatory sites in ruminants. They represent a relatively high proportion of peripheral blood in ruminants (15–60% of PBMC), especially in young individuals, suggesting an important role in host defense [28]. It has been found that gamma delta T cells can produce cytokines such as IFN-γ, IL-17, IL-10 and TGFβ, which can induce cytotoxic activity and memory response and are important for controlling pathogenic infections [29]. 

Cytokines are polypeptide factors that are secreted or released in multiple ways and exert autocrine and/or paracrine effects. They are pleiotropic and usually control cell growth, survival, and/or differentiation, making them key regulators of the immune system [30–32]. IFN-γ is a critical Th1 cytokine that defends against infection and is primarily produced by activated T cells and natural killer cells [33]. As a pleiotropic anti-inflammatory cytokine, IL-4 plays an important role in antitumor, anti-infection, and other disease prevention mechanisms [34]. IL-17A is one of the six members of the IL-17 cytokine family; it is a multifunctional cytokine that regulates both innate and adaptive immune responses [35]. This study detected three major representative cytokines, IFN-γ, IL-4, and IL-17A, in PBMCs stimulated with different polyclonal stimulators using different immunological techniques. We observed differences in the amounts of cytokines expressed by different individuals and different cell populations. After sheep were infected with *Mycoplasma agalactiae*, the expression of IFN-γ in CD4^+^ and CD8^+^ T cells significantly increased compared with that in uninfected controls [36]. Studies on the detection of cytokines, such as IFN-γ and IL-4 have shown that cellular immune responses and cytokines reduce sheep viremia [37]. PBMCs from sheep immunized with *Fasciola hepatica* antigens generated high levels of IFN-γ and were re-stimulated in culture [7]. The detection of IL-17A in sheep and goats indicated that IL-17A is mainly expressed by CD4^+^ and CD8^+^ T cells [38].

We used two polyclonal stimulators, PMA/Ionomycin and Con A, to stimulate PBMC cytokine production. We found that PMA/Ionomycin stimulation increased the output of the three cytokines more significantly comparing with Con A stimulation. PMA/Ionomycin, polyclonal and nonspecific reagents, induce signal transduction and produce cytokines. PMA, an analog of a diglyceride, is a primary mediator of many intracellular signaling pathways [39]. Ionomycin stimulates the endoplasmic reticulum to release Ca^2+^, activates Ca^2+^-sensitive enzymes, and interacts with PMA [40]. Concanavalin A (Con A) is a polyclonal mitogen that induces acute liver injury by activating T lymphocytes [41]. These two compounds have been widely used to stimulate the activation and proliferation of T lymphocytes [5, 7, 42]. PMA/Ionomycin is typically used in flow cytometry research, whereas Con A is often used as a polyclonal stimulator in ELISA research. Cell proliferation is a fundamental feature of the adaptive immune response, which can occur through the stimulation of cultured cells. The division-tracking dye carboxyfluorescein diacetate succinimidyl ester (CFSE) can be used to monitor the number of cell divisions. We used Con A to stimulate PBMCs and analyzed the proliferation of T lymphocytes using CFSE. The proliferation of lymphocytes, CD4^+^ T cells, and CD8^+^ T cells increased gradually with the prolongation of ConA stimulation time. In addition to the two stimulators used in our study, other polyclonal stimulators are often used in studies to stimulate T cell activation and proliferation. Phytohemagglutinin (PHA) is commonly used as a mitogenic lectin and is widely used to stimulate T cells. A physiologically relevant approach used beads coated with anti-CD3 and anti-CD28 to stimulate T cells in a manner that partially mimics antigenic stimulation [43]. In a related study on the primary porcine spleen, both PHA and Con A effectively stimulated T cell proliferation and IL-2 production [44]. Stimulating PBMCs with anti-CD3/CD28 coated beads provides an alternative method for driving T cell expansion that may be very useful in immunotherapy [45]. We can thus select appropriate T cell polyclonal stimulants depending on the experimental design and experimental purpose.

## Conclusion

This study is the first systematic measurement of the fundamental immune indicators of healthy sheep T lymphocytes worldwide, providing meaningful references for relevant scientific researchers. This can help us evaluate the immune response of sheep to pathogens and improve sheep welfare.

## Methods

### Animals

Thirty-six healthy female sheep, 4‒6 months of age, were raised in helminth-free conditions (*Brucella* negative in serum, Table 1), fed hay and threshing corn, and provided clean water ad libitum in pens. Daily observations and physical examinations were conducted to evaluate the health status of each sheep throughout the trial.

**Table 1:** Basic information of sheep in this study

No	Gender	Weight(Kg)	*Brucella*
1	Female	25.2	-
2	Female	22.4	-
3	Female	20.4	-
4	Female	27.5	-
5	Female	22.7	-
6	Female	22.8	-
7	Female	23.8	-
8	Female	20.4	-
9	Female	23.0	-
10	Female	21.1	-
11	Female	21.0	-
12	Female	23.8	-
13	Female	21.9	-
14	Female	25.7	-
15	Female	26.0	-
16	Female	22.4	-
17	Female	25.7	-
18	Female	25.8	-
19	Female	23.8	-
20	Female	18.2	-
21	Female	23.2	-
22	Female	19.8	-
23	Female	19.8	-
24	Female	18.3	-
25	Female	30.5	-
26	Female	22.7	-
27	Female	24.3	-
28	Female	24.0	-
29	Female	23.5	-
30	Female	30.5	-
31	Female	20.9	-
32	Female	19.0	-
33	Female	24.0	-
34	Female	24.7	-
35	Female	21.2	-
36	Female	18.0	-

### PBMC isolation

Plasma was aliquoted after heparin sodium anti-coagulated jugular venous blood was centrifuged at 1000 × *g* for 10 min. PBMCs were acquired by density-gradient centrifugation, but without buffy-coat stage [46]. Instead, whole blood was diluted 1:1 in sterile phosphate buffer saline (PBS), mixed, layered on an equal volume of lymphocyte separation media (Lymphoprep 1.087 g/L, TBD Science, Wuhan, China), and centrifuged at 1130 × *g* at 22 °C for 30 min with no break. The white PBMC layer was collected and washed twice with PBS. The cells were counted and diluted to a concentration of 1 × 10^6^/mL in RPMI-1640 medium supplemented with 10% FBS (Gibco, Waltham, MA), 2 mM l-glutamine, 100 IU/mL penicillin, 50 μg/mL streptomycin, and 50 µM beta-mercaptoethanol (Sigma-Aldrich, MO, USA).

### PBMC stimulation

PBMCs were diluted to 1 × 10^6^/mL in RPMI-1640 medium. For flow cytometry, 1 mL of cell suspension was added to 12 × 75- mm round-bottom tubes plus PMA (20 ng/ml)-Ionomycin (1 μg/ml)-BFA (1 μg/ml) or medium with BFA (1 μg/ml). The cells were collected for flow cytometry after 4 h of stimulation at 37 °C and 5% CO_2_. For ELISAs, 200 µL of cell suspension was added to U-bottom cell culture plates (Corning, NY, USA) with Con A (5 μg/ml), PMA (20 ng/ml) + Ionomycin (1 μg/ml), or only medium. The culture supernatants were collected after 24 h for cytokine analysis. For ELISpot analysis, 200 µL of cell suspension was added to the ELISpot plates plus Con A (5 μg/ml), PMA (20 ng/ml) + Ionomycin (1 μg/ml), or only medium. The plate was incubated for 24 h before spot detection. The experiment was conducted in triplicate.

### Cytokine ELISAs

IFN-γ, IL-4, and IL-17A levels in culture supernatants were quantified using commercial ELISA kits (Bovine IFN-γ/IL-4/IL-17A ELISA^BASIC^ kit, Mabtech, Nacka, Sweden). Each sample was tested in triplicate. The assays were read using a Multiskan SkyHigh Microporous plate spectrophotometer (Thermo Fisher Scientific, MA, USA) at 450 nm, and standard curves were generated to quantify the antibody and cytokine levels.

### ELISpot assays

The spots produced by the lymphocytes were detected using a Bovine IFN-γ/IL-4 ELISpot^PLUS^ kit (Mabtech). The cells were removed by emptying the plates. The plates were washed 5 times with PBS and incubated for 24 h. The detection antibody was diluted in PBS-0.5% fetal calf serum (FCS), incubated for 2 h at room temperature (RT), diluted with streptavidin-HRP (1:1000) in PBS-0.5% FCS, and incubated for 1 h at RT. The TMB substrate solution was added in the dark until distinct spots emerged. Color development was stopped with deionized water. Each sample was tested in triplicate. The spots were counted using an ELISpot reader system (AID, Straßberg, Germany) after drying the plate.

### Flow cytometry

PBMCs were collected, washed twice, stained with mouse anti-sheep CD4 and mouse anti-sheep CD8 antibodies (AbD Serotec, Oxford, UK) for 30 min at 4 °C in the dark, and then washed in staining buffer (PBS + 0.1% bovine serum albumin (BSA) + 0.05% sodium azide). For intracellular staining, PBMCs were fixed in 4% paraformaldehyde for 8–10 min, washed in staining buffer, permeabilized in staining buffer containing 0.1% saponin, and incubated at 4 °C overnight. After washing, PBMCs were stained with mouse anti-bovine IFN-γ, mouse anti-bovine IL-4, mouse anti-human IL-17A (which cross-reacts with sheep) [47] in staining buffer supplemented with 0.1% saponin for 30 min at 4 °C in the dark and washed in staining buffer. Acquisition was performed on a BD FACSCelesta flow cytometer (Becton Dickinson, NJ, USA), and data were analyzed using FlowJo software (Becton Dickinson). Unstimulated cells were used as the negative control, and fluorescence minus one (FMO) control was used as the gating strategy.

### Proliferation assays

PBMCs (1 × 10^7^/mL) were washed with pre-warmed PBS-0.1% BSA and resuspended with 5 μM carboxyfluorescein diacetate succinimidyl ester (CFSE, Invitrogen, CA, USA) [48]. The cells were quenched by diluting with 6 volumes of RPMI-1640 medium supplemented with 10% FBS for 5 min at 4 °C, then washed in the same manner two more times with complete RPMI-1640 medium. CFSE-labeled cells were stimulated with Con A (5 μg/ml) or medium control for 72, 96, or 120 h. At these times, cells were collected and washed twice, stained with mouse anti-sheep CD4 and mouse anti-sheep CD8 antibodies for 30 min at 4°C in the dark, then washed in staining buffer. Samples were assayed using a FACSCelesta flow cytometer (Becton Dickinson) and data were analyzed using FlowJo software (Becton Dickinson).

### Statistical analysis

Analyses were performed using the GraphPad Prism 8.0 software (GraphPad Software Inc., USA). Comparisons were performed using the unpaired Student’s *t*-test for comparing two groups and one-way ANOVA for three or more groups. Differences were considered statistically significant at *p < 0.05, **p < 0.01, ***p < 0.001, ****p < 0.0001. Data are expressed as means ± standard deviation (SD).

## Data Availability

All data generated or analyzed during this study are included in this article.

## Abbreviations

PBMC: Peripheral blood mononuclear cell
PBS: Phosphate buffer saline
BSA: Bovine serum albumin
CD: Cluster of differentiation
IFN-γ: Interferon-gamma
IL: Interleukin
ELISA: Enzyme-linked immunosorbent assay
ELISpot: Enzyme-linked immunospot assay
SFC: Spot-forming cells
FCS: Fetal calf serum
RT: Room temperature
FMO: Fluorescence minus one
Con A: Concanavalin A
PMA: Phorbol-12-myristate-13-acetate
BFA: Brefeldin A
PHA: Phytohemagglutinin
Th: Helper T cell
CFSE: Carboxyfluorescein succinimidyl ester
